# A Cardiac Pseudoaneurysm as a Thromboembolic Source: Acute Visual Loss due to Cardiac Emboli

**DOI:** 10.1155/2020/3192957

**Published:** 2020-03-11

**Authors:** Fernando Montenegro Sá, Sara I. L. Fernandes, Rita J. R. Carvalho, Luís M. G. Santos, José A. S. Antunes, Joana M. Guardado, João C. A. Morais

**Affiliations:** Cardiology Department, Leiria Hospital Center, Portugal

## Abstract

Acute visual loss is rarely caused by a heart condition. This manuscript transcribes a case report of a 36-year-old patient with a 2-year history of aortic valve replacement due to bicuspid aortic valve endocarditis that presents to the emergency department with an acute right eye visual loss. After ophthalmologic investigation identified a central retinal artery occlusion, a transthoracic echocardiography was performed to search for a possible cardiac embolus, despite the patient presenting INR values of 2-2.5 for the last year. A mitral-aortic intervalvular fibrosa pseudoaneurysm was identified. A transoesophageal echocardiography was then performed, identifying a small clot logged inside the pseudoaneurysm that protruded to the left ventricle outflow tract. After INR-adjusted warfarin treatment to levels between 3 and 4, the pseudoaneurysm was surgically closed. This is a rare case since the likely source of embolism to the central retinal artery was the thrombus logged inside the pseudoaneurysm despite a standardly accepted therapeutic INR.

## 1. Introduction

The mitral-aortic fibrosa (MAF) consists of a thin layer of fibrous avascular tissue that separates the anterior mitral leaflet from the posterior portion of the aortic root [1]. It is limited by the left atrium (posterolaterally), the left ventricular outflow tract (LVOT, inferiorly), and the pericardium (superiorly). It is prone to infection, and, as a result, a pseudoaneurysm (PMAF) may develop—an uncommon condition that typically consists in a pulsatile cavity in the mitral-aortic junction communicating with the left ventricular outflow tract (LVOT) [1–3]. It is most frequently caused by aortic valve endocarditis, particularly in prosthetic valves [1, 4]. It may be identified by transthoracic echocardiography (TTE) and transoesophageal echocardiography (TOE) in postcardiac surgery asymptomatic patients or may present itself with many unspecific symptoms: the current literature describes fever and active signs of endocarditis in nearly 40% of PMAF patients and heart failure signs in 16%. Only 12% presents with cerebrovascular or embolic events [2]. It may be a severe condition if complete rupture occurs [5], but treatment is available through surgical or percutaneous closure [2, 4, 6].

## 2. Case Presentation

### 2.1. History of Presentation

A 36-year-old male presented to the emergency department with complaints of acute (approximately 1-hour evolution) and painless visual loss of the right eye. He was promptly observed by ophthalmology that determined a fully preserved (6/6) visual acuity of the left eye but near blindness of the right eye (6/60). The patient was agitated but had no other complaints. At inspection, the eye had no signs of infection or trauma. Neurological exam did not identify any change: sensory and motor examinations were normal and there was no facial asymmetry, aphasia, dysarthria, or dysmetria. Oculomotor movements and pupillary reflexes were preserved.

### 2.2. Past Medical History

Previous medical history included a known bicuspid aortic valve disease. 2 years later, and after a dental procedure, the patient developed an aortic valve endocarditis due to an infection from S*treptococcus sanguinis*. This led to an ischemic stroke, from which the patient recovered completely. He was under antibiogram-directed antibiotic therapy for 1 month when a second stroke happened, and he was then submitted to an urgent surgical replacement of the aortic valve for a mechanical prosthesis that went well. He was discharged with a transthoracic echo referring a normofunctional prosthesis with no other significant alterations and an indication for warfarin therapy for INR target between 2 and 3. At the time of presentation of this case, the patient had medical records available that confirmed INR values between 2 and 2.5 for the last year.

### 2.3. Differential Diagnosis

Differential diagnosis for single eye acute visual loss includes an acute stroke/transient ischemic accident, compressive lesion, trauma, infection, Purtscher retinopathy and occlusion of the central retinal artery due to either atherosclerotic disease, embolism, arteritis or the presence of a hypercoagulable state (due to neoplastic disease or inherited pro-coagulative diseases, for example) [7].

### 2.4. Investigations

**Figure 1:**
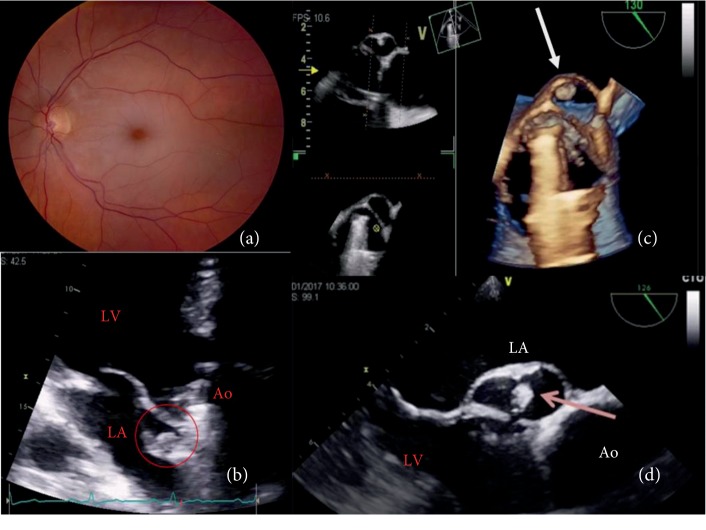
(a) Cherry red spot at the center of the fundoscopy. (b) Echolucent area in the mitral-aortic fibrosa with a hyperechogenic structure inside (red circle). (c) Reconstruction through transoesophageal echocardiography showing the mitral-aortic intervalvular fibrosa with a pseudoaneurysm (arrow) communicating with the left ventricle outflow tract and a free thrombus inside. (d) Transoesophageal echocardiography at 126° showing a free hyperechogenic highly mobile structure inside a pseudoaneurysm in the mitral-aortic intervalvular fibrosa, protruding into the LVOT through a small orifice. LA: left atrium; LV: left ventricle; Ao: ascending aorta.

**Figure 2:**
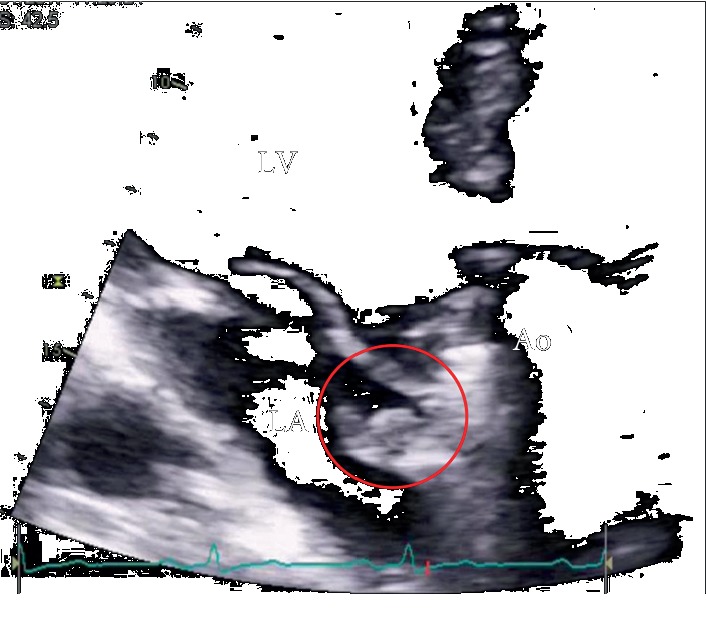
Echolucent area in the mitral-aortic fibrosa with a hyperechogenic structure inside (red circle). LA: left atrium; LV: left ventricle; Ao: ascending aorta.

**Figure 3:**
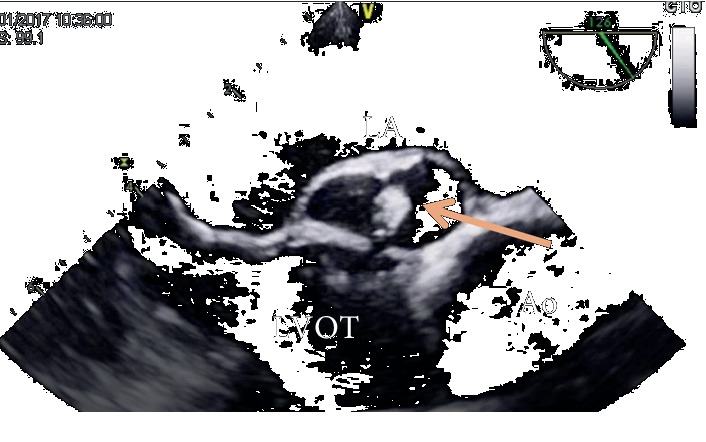
Transoesophageal echocardiography at 126° showing a free hyperechogenic highly mobile structure inside a pseudoaneurysm in the mitral-aortic intervalvular fibrosa, protruding into the LVOT through a small orifice. LA: left atrium; LVOT: left ventricle outflow tract; Ao: ascending aorta.

**Figure 4:**
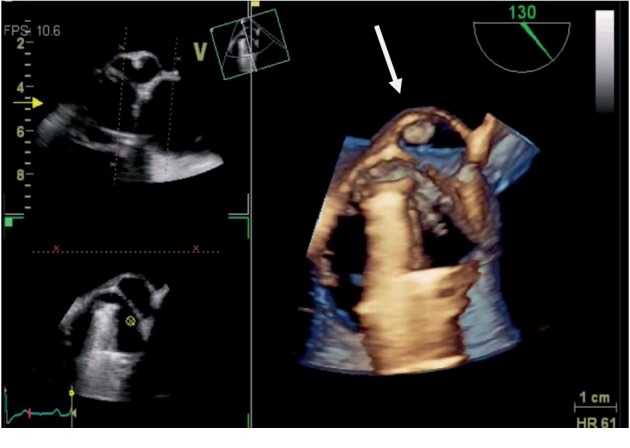
3D reconstruction through transoesophageal echocardiography showing the mitral-aortic intervalvular fibrosa with a pseudoaneurysm (arrow) communicating with the left ventricle outflow tract and a free thrombus inside.

A fundoscopic exam was immediately performed and identified the foveola assuming a cherry red spot-like image with retinal whitening, which suggested that the visual loss had been caused by a central retinal artery occlusion (Figure 1(a)). Due to the patient's previous medical history, cardiology observation was required to search for a cardiac source of embolism. The patient's physical exam was normal, with cardiac auscultation identifying a clear metallic click as the second cardiac sound and a slight nonradiating systolic murmur (I/VI). A TTE was performed, showing a nonobstructive aortic prosthesis and a periprosthetic echolucent pulsatile region with a hyperechogenic mobile round structure inside (Figures 1(b) and 2). To better characterize these findings, a TOE was performed (Figures 1(c), 1(d), 3, and 4) revealing a free hyperechogenic highly mobile structure suggestive of a free thrombus inside a pseudoaneurysm in the mitral-aortic intervalvular fibrosa, protruding into the left ventricular outflow tract through a small communication orifice (supplementary material [Supplementary-material supplementary-material-1] and [Supplementary-material supplementary-material-1]).

### 2.5. Treatment

In the acute setting in the emergency department, treatment consisted of the intermittent digital massage of the affected eye and the use of ocular hypotensive drugs and intravenous acetazolamide. The patient had a partial recovery of right eye visual acuity to 6/12. After 3 months of INR-adjusted warfarin treatment for target 3.0–3.5, the patient was submitted to a surgical pseudoaneurysm closure with a synthetic patch.

### 2.6. Outcomes

Since surgical pseudoaneurysm closure, and after the 1-year follow-up, the patient had no other embolic event. Visual acuity had no further improvements.

## 3. Discussion

This case represents a scenario of a postcardiac surgery complication with a very rare clinical presentation. In our understanding, the PMAF created a low-flow condition that, despite an INR between 2 and 2.5, still allowed a thrombus formation. The embolization of this small thrombus was the highly probable cause for the central retinal artery occlusion that caused the patient's symptoms. Regarding ophthalmological investigation, and after fundoscopy identification of retinal whitening with a macular cherry red spot, no further tests are indicated [7].

A review of all relevant English language articles published until 2010 identified 88 PMAF cases in the literature: only 11 patients presented with a cerebrovascular or an embolic complication. Of these, a clot was identified inside the pseudoaneurysm in only 5 cases [2]. Stroke due to a thrombus inside the PMAF was previously described, but to our knowledge, this is the first described case of central retinal artery occlusion.

Acute visual loss is rarely due to a cardiac cause, but in patients with high embolic risk conditions, it should be systematically thought of, since diagnosis may be difficult [5] but specific adequate treatment is available [6]. In this case, the final diagnosis was a cardioembolic central retinal artery occlusion, and most patients continue to experience severe vision loss during follow-up despite the current standard therapy [8].

This case highlights that in patients with a high embolic risk, the search for a cardiac embolism after any possible embolic event must be thorough. According to guidelines and recommendations, TTE is the first tool, and sometimes a TOE is also required [3]. This report represents a clinical scenario where a rare extracardiac manifestation (visual loss) was the event that prompted an exhaustive investigation that allowed surgical correction for the underlying problem.

## Supplementary Materials

Supplementary 1 Video 1: transoesophageal echocardiography at 126° showing the mitral-aortic intervalvular fibrosa with a pseudoaneurysm and a highly mobile hyperechogenic mass suggestive of a small thrombus.

Supplementary 2 Video 2: transoesophageal echocardiography with 3D reconstruction showing a highly mobile mass (the thrombus) protruding through the ostium of the pseudoaneurysm to the left ventricular outflow tract.
